# Advanced Proteomic Approaches to Elucidate Somatic Embryogenesis

**DOI:** 10.3389/fpls.2018.01658

**Published:** 2018-11-20

**Authors:** Victor Aguilar-Hernández, Víctor M. Loyola-Vargas

**Affiliations:** ^1^Catedrático CONACYT, Unidad de Bioquímica y Biología Molecular de Plantas, Centro de Investigación Científica de Yucatán, Mérida, Mexico; ^2^Unidad de Bioquímica y Biología Molecular de Plantas, Centro de Investigación Científica de Yucatán, Mérida, Mexico

**Keywords:** 2D electrophoresis, differentiation, plant growth regulators, proteomics, somatic embryogenesis

## Abstract

Somatic embryogenesis (SE) is a cell differentiation process by which a somatic cell changes its genetic program and develops into an embryonic cell. Investigating this process with various explant sources *in vitro* has allowed us to trace somatic embryo development from germination to plantlets and has led to the generation of new technologies, including genetic transformation, endangered species conservation, and synthetic seed production. A transcriptome data comparison from different stages of the developing somatic embryo has revealed a complex network controlling the somatic cell’s fate, suggesting that an interconnected network acts at the protein level. Here, we discuss the current progress on SE using proteomic-based data, focusing on changing patterns of proteins during the establishment of the somatic embryo. Despite the advanced proteomic approaches available so far, deciphering how the somatic embryo is induced is still in its infancy. The new proteomics techniques that lead to the quantification of proteins with different abundances during the induction of SE are opening this area of study for the first time. These quantitative differences can elucidate the different pathways involved in SE induction. We envisage that the application of these proteomic technologies can be pivotal to identifying proteins critical to the process of SE, demonstrating the cellular localization, posttranslational modifications, and turnover protein events required to switch from a somatic cell to a somatic embryo cell and providing new insights into the molecular mechanisms underlying SE. This work will help to develop biotechnological strategies for mass production of quality crop material.

## Introduction

**FIGURE 1:**
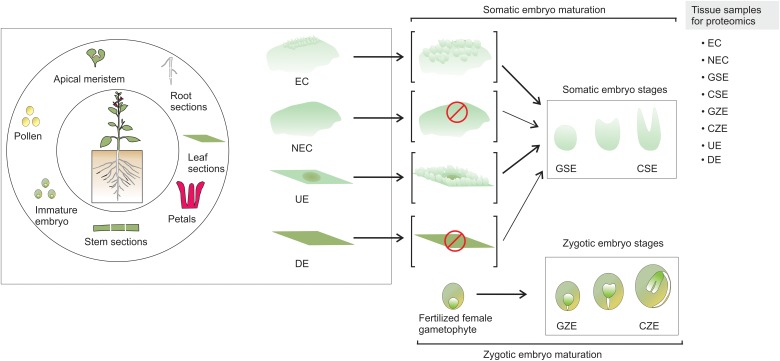
Somatic embryogenesis. SE can be induced from a wide variety of plant parts. There are two known pathways to produce somatic embryos. The explant produces embryogenic callus, and somatic embryos emerge from the embryogenic mass. In the second case, the somatic embryos arise directly from the explant, without the formation of embryogenic mass. EC, embryogenic callus; NEC, non-embryogenic callus; GSE, globular somatic embryo; CSE, cotyledonar somatic embryo; GZE, globular zygotic embryo; CZE, cotyledonar zygotic embryo; UE, undifferentiated explant; DE, differentiated explant.

Somatic embryogenesis (SE) is of high significance to studies on plant development, particularly the changes that occur from the early to mature embryonic stages, genetic transformation of various plant species, endangered species conservation, and synthetic seed production (Loyola-Vargas and Ochoa-Alejo, 2016). SE is a process by which a unique somatic cell or a cluster of cells changes, following a differentiation program, into an embryo, which is then converted by consecutive development stages into an adult plant (Yang and Zhang, 2010). It is accepted that during this process the somatic cell reverts back into an embryogenic cell (Sugimoto et al., 2011). A variety of explant tissues, which include pollen, apical meristem, root, stem, and leaf sections, as well as immature embryos, have been used to induce SE (Figure 1).

In contrast to non-embryogenic callus (NEC) and differentiated explant (DE) that does not yield somatic embryos, the embryogenic callus (EC) and undifferentiated explant (UE) tissue contain embryogenic cells generated from the somatic cells, which are converted through a path from globular somatic embryo (GSE) to cotyledonar somatic embryo (CSE) into a mature embryo that, after a germination process, can generate a plantlet. It is accepted that a fertilized female gametophyte follows a comparable morphological path to SE, from a globular zygotic embryo (GZE) to cotyledonar zygotic embryo (CZE) (Loyola-Vargas and Ochoa-Alejo, 2016).

Somatic embryogenesis implies a switch from a somatic to an embryogenic state that is able to develop into a mature plant. These changes involve coordinated cellular, biochemical, genetic, and epigenetic changes produced by exogenous plant growth regulators (PGRs) or stress (i.e., mechanical damage or wounding), which trigger a massive expression of genes in several waves of expression. The first wave of expressing genes comprises transcription factors (TFs) such as *AGL15*, *LEC2*, *LEC1*, *BBM*, *MAD*-box, and *WUS* (Yang et al., 2012; Indoliya et al., 2016; Cao et al., 2017; Jamaluddin et al., 2017; Magnani et al., 2017). Then a second wave of transcription involves genes coding for enzymes involved in the homeostasis of auxins and cytokinins, as well as other PGRs (Ayil-Gutiérrez et al., 2013; Márquez-López et al., 2018).

More recently, attention has shifted to unveiling how the somatic cell proteome changes to promote the development of an embryogenic cell, maturation, and germination of the somatic embryo. This is not a trivial task and represents a significant research challenge, since TFs are low abundance proteins. The task is complicated by crosstalk among different metabolic pathways during the induction of SE.

In this review, we describe the currently available technologies for proteomics studies, and in turn discuss the current progress of these technologies’ use for the study of SE and the changing protein patterns during the establishment of the somatic embryo.

## Plant Proteomics Technologies

The development of different proteomics technologies has led, during the last two decades, to advances in the identification of proteins involved in the induction of SE. In brief, a proteomic pipeline consists of collecting the suitable tissue, protein homogenate preparation, proteolytic digestion, peptide separation, detection by MALDI-TOF or LC-MS/MS, and data processing. Most of the proteomic studies on SE are based on protein extracts derived from contrast morphological stages tissue, from either explant or calli to matured embryo, distinguished by a relatively easy and tractable feature such as color and size, shape, or arrange of the cells. Collectively, those studies have releveled essential and even unique proteins of somatic embryo stages. However, the precise collection of proteins triggering the conversion and fate of a somatic cell to a somatic embryo is an enigma. As it is dependent on multiple factors including different types of stress, media composition, genotype, and even the origin of a somatic cell (Quiroz-Figueroa et al., 2002; Yang and Zhang, 2010; Campos et al., 2017), likely multiple proteins could sustain the somatic to embryogenic cell conversion. Those proteins could be low abundance proteins or expressed in a spatiotemporal-dependent fashion, thus limiting their detection in complex samples presented as crude protein homogenate. The implementation of protein fractionation or enrichment before or after trypsin digestion could help to overcome the limitation by reducing the complexity of the sample for the mass spectrometry protein identification. A sample with a reduced complexity via an antibody-based enrichment for acetylated peptides from *Picea asperata* somatic embryos had facilitated the generation of deep acetylome that contains nearly two acetylated sites per protein identified (Xia et al., 2016).

Deciphering the interconnected proteome responsible for somatic cell switch to somatic embryo requires the determination of important protein features such as protein location, protein stability, posttranslational modifications (PTMs), and protein-protein interactions for the proteins present in the proteome of the SE process. Besides keeping most of the proteins in the sample in solution during the preparation of protein homogenate or in step-wise isolation of specific kinds of proteins (Niu et al., 2018), the preservation of proteins and PTM is a key factor to get insights into SE proteome (Peltier et al., 2004; Xia et al., 2016; Aguilar-Hernández et al., 2017).

**FIGURE 2:**
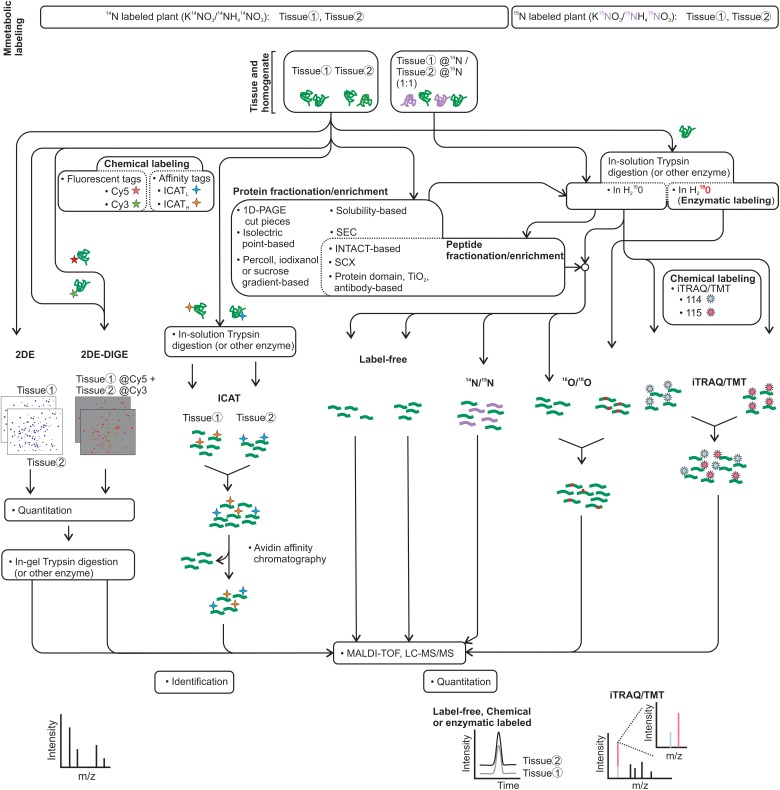
Proteomic pipeline approaches for the SE process. 2D and 2D-DIGE that use fluorescent tags for the protein followed by MALDI-TOF or LC-MS/MS have been used extensively to contrast protein samples from the SE process. Label-free and iTRAQ, which label peptides with an isobaric tag for quantification, have been used in a few studies. The chemical labeling of proteins by ICAT reagents and peptides by TMT reagents, the metabolic labeling by ^15^N *in vivo* or by ^18^O *in vitro* at the trypsin digestion, and the exploitation of protein or peptide fractionation/enrichment in the proteomic approaches may facilitate SE discoveries. Identification by MS/MS and quantification by either as a signature in extracted ion chromatogram (XIC) or reporter ions are indicated.

**FIGURE 3:**
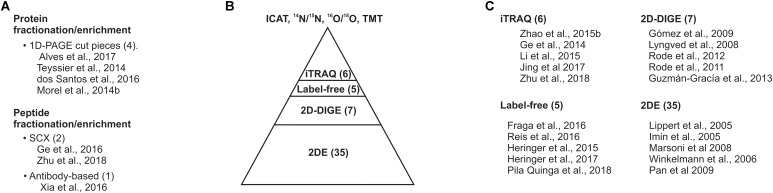
Gel-based proteomic approaches dominate the proteomic studies on SE. **(A)** Protein or peptide sample fractionation/enrichment. **(B)** Current proteomic approaches implanted for surveying the SE proteome. **(C)** Top five cited studies on SE proteomics. The number within prentices indicates the number of studies compiled in this review.

The protein homogenate can be subjected to a variety of proteomic approaches such as (i) two-dimensional gel electrophoresis (2DE), (ii) 2DE differential in-gel electrophoresis (2DE-DIGE), (iii) label-free proteomics, (iv) isotope-coded affinity tagging (ICAT), various isotope-labeling methods used for quantitative proteomics, including ^14^N/^15^N, ^16^O/^18^O, or (v) iTRAQ and TMT, isobaric tags exploited for quantitative proteomics (Figure 2). Each approach has advantages and disadvantages that can be complemented by the implementation of multiple strategies in parallel. Most of the SE proteomic studies were based on 2DE and 2DE-DIGE. Overall, each of them revealed the identity of nearly 100 proteins of the 2,000 proteins that could be detected and quantified in the gels (Figure 3). 2DE-DIGE eliminates much of the variation between gels observed in 2DE since two samples are resolved at the same time in the same gel, but requires special equipment for the image capture-laser scanning of the gels. The label-free proteomics is a gel-free approach and has positioned itself as the cheapest one proteomic approach, identifying over a thousand proteins; however, it requires intensive bioinformatics if the number of samples to compare is higher than two samples. The iTRAQ- and TMT-based quantitative proteomic approaches lead to the comparison of up to 10 samples at the same time; however, they are relatively expensive compared to the other quantitative proteomic methods. The ^14^N/^15^N approach reciprocally mixes the subject samples early, thus eliminating any further effect during protein homogenate processing, but their data analysis cannot be trivial. Recently, it has been shown that it is possible to achieve the metabolic labeling of proteins by replacing any source of nitrogen in the plant cell culture media, hydroponic solution or soil with heavy nitrogen, ^15^KNO_3_, and ^15^NH_4_^15^NO_3_ (Ippel et al., 2004; Kim et al., 2005; Engelsberger et al., 2006; Benschop et al., 2007; Huttlin et al., 2007; Lanquar et al., 2007; Nelson et al., 2007; Bindschedler et al., 2008; Hebeler et al., 2008; Schaff et al., 2008; Laganowsky et al., 2009; Stanislas et al., 2009; Figure 2). ^15^N metabolic labeling proteins have displayed at least 98% and no phenotypic differences observed between metabolically labeled and unlabeled plants (Nelson et al., 2007). ^15^N have not been used in SE studies and its future implementation could help to get insights into quantifying proteins with a role in the SE process.

The integration of fractionation and/or type-enrichment for proteins or peptides, including resolving protein homogenate by 1D-PAGE followed by in-gel digestion and LC-MS/MS antibody-based acetylated-lysine enrichment (Zhao et al., 2015a,b; dos Santos et al., 2016; Xia et al., 2016; Alves et al., 2017), may provide new insights and in-depth proteome coverage during the induction of SE. However, few of the exquisite variety of proteomic tools have been implemented so far.

Since many of the early cell events, during the induction of SE, have been associated with nuclei and metabolic pathways compartmentalizing in multiple organelles, techniques such as Percoll, iodixanol, or sucrose gradient-dependent, and INTACT (Isolation of Nuclei Tagged in specific Cell Types)-dependent cell organelles fractionation can result in promissory for SE protein dynamic studies (Dunkley et al., 2004; Kleffmann et al., 2004; Olinares et al., 2010; Deal and Henikoff, 2011; Sikorskaite et al., 2013).

The important effect of auxin during the SE process is evident, and given that its perception and signaling are mediated by ubiquitination, a PTM with ubiquitin, there is promise in exploring ubiquitination events and their role in SE biology. PTM can be determined by an enrichment at the protein or peptide level after trypsin digestion with a protein domain, compounds (i.e., TiO_2_), and antibody-based affinity chromatography resulting in the identification of a considerable number of proteins with PTMs and the modified sites (Thingholm et al., 2006; Kim et al., 2013; Aguilar-Hernández et al., 2017). Multiple tags have been developed to perform quantitative proteomics either at the protein level such as ICAT or at the peptide level ITRAQ/TMT.

A considerable number of proteomic studies on SE have been performed, on the basis of samples from contrasting phenotypes such as EC, NEC, UE, and DE, as well as comparison of the somatic embryo stages NEC, UE, or ZE in various plant species (Table 1). A variety of plant species’ proteomes have been surveyed, mainly focusing on deciphering the SE process. Crops are the dominant group in the SE proteomes reported so far. It is not a surprise that plant models with substantially advanced genetic tools such as *Arabidopsis thaliana* do not dominate these studies, since somatic embryo-based transformation is rare.

**Table 1:** Embryogenic vs. non-embryogenic and non-embryogenic vs. somatic embryogenic, comparative proteomic studies from a variety of plant models.

Species	Explant	Proteomic approach	Reference	Protein classes identified^∗^
**EC vs. NEC**				
*Cyphomandra betacea*	Leaf sections and immature zygote embryo	2DE, LC-MS/MS, MASCOT	Correia et al., 2012b	• ROS scavengers • Metabolic enzymes • Primary metabolism associated enzymes • Fatty acid biosynthesis/metabolism enzymes • Glycolysis • Molecular chaperones • PGR metabolism/signaling pathways • Polyamine metabolic pathway • Signal transduction molecules
*Persea americana*	Immature zygote embryo	2DE-DIGE followed by MALDI-TOF	Guzmán-García et al., 2013	
*Zea mays*	Immature zygote embryo	2DE LC-MS/MS Q-TOF, IDENTITYE	Varhaníková et al., 2014	
		iTRAQ, SCX chromatography, LC-MS/MS, MASCOT, WGCNA	Ge et al., 2017	
*Gossypium hirsutum*	Hypocotyl sections	iTRAQ, SCX chromatography, LC-MS/MS, Proteome Discoverer-MASCOT	Zhu et al., 2018	
*Musa* spp. AAA cv. Grand Naine	Immature male flower buds	2DE, MALDI-TOF, MASCOT	Kumaravel et al., 2017	
*Solanum betaceum*	Leaf sections	1D, MALDI-TOF, MASCOT	Alves et al., 2017	• Proteolytic enzymes • Proteasomal components/factors • ROS scavengers • Primary metabolism associated enzymes • Molecular chaperones • Glycolysis • ATP synthesis • Defense-related proteins • Cytoskeleton • Transcriptional regulation • Signal transduction molecules
*Fraxinus mandshurica*	Immature zygote embryo	2DE, MALDI-TOF, MASCOT	Liu et al., 2015	
*Vitis vinifera*	Leaf sections	2DE, LC-MS/MS, TurboSEQUEST	Marsoni et al., 2008	
*Cyclamen persicum*	Unpollinated ovules	2DE-DIGE, MALDI-TOF, MASCOT	Lyngved et al., 2008	
*Crocus sativus*	Corm meristems	2DE, MALDI-TOF, MASCOT	Sharifi et al., 2012	
*Larix principis-rupprechtii*	Immature zygote embryo	1D cut pieces, iTRAQ, LC-MS/MS, Proteome Discoverer-MASCOT	Zhao et al., 2015b	
*Zea mays*	Immature zygote embryo	2DE, MALDI-TOF, MASCOT	Sun et al., 2013	
*Vitis vinifera*	Anther tissue	2DE followed by MALDI-TOF, MASCOT	Zhang et al., 2009	• Disease/defense-related proteins • Membrane trafficking • Nutrient response-related proteins • Cytoskeleton • Primary metabolism associated enzymes • ROS scavengers • Glycolysis • Transcriptional regulation • Cell wall synthesis proteins • Calcium signaling related proteins • Cell cycle • Signaling transduction molecules • Molecular chaperones
*Araucaria angustifolia*	Immature zygote embryo	1D cut pieces, LC-MS/MS, MaxQuant, MaxLFQ	dos Santos et al., 2016	
*Pinus nigra*	Immature zygote embryo	2DE, MALDI-TOF, ProteinScape-MASCOT	Klubicová et al., 2017	
**SE vs. GE**				
*Quercus suber*	Immature zygote embryo	2DE-DIGE, MALDI-TOF, MASCOT	Gómez et al., 2009	• Primary metabolism associated enzymes
**SE stages comparison**				
*Citrus sinensis*	Embryogenic line	2DE, MALDI-TOF, MASCOT	Pan et al., 2009	• Glycolysis • ROS scavengers • Molecular chaperones • Signal transduction molecules • Polyamine metabolic pathway • Disease/defense-related proteins • Proteasomal components/factors
*Elaeis guineensis*	Zygote embryos	2DE, MALDI-TOF, MASCOT	Silva et al., 2014	
*Quercus suber*	Immature zygote embryo	2DE-DIGE, MALDI-TOF,	Gomez-Garay et al., 2013	
*Picea glauca*	Embryogenic line 11026	2DE, LC-MS/MS, MASCOT	Lippert et al., 2005	• Glycolysis • ROS scavengers • Molecular chaperones • Proteasomal components/factors • Storage proteins
*Cyclamen persicum*	Embryogenic suspension culture	2DE-DIGE, LC-MS/MS, MASCOT	Rode et al., 2012	
*Larix principis-rupprechtii*	Immature zygote embryo	1D cut pieces, iTRAQ, LC-MS/MS, Proteome Discoverer-MASCOT	Zhao et al., 2015a	• Glycolysis • ROS scavengers • Molecular chaperones • Disease/defense-related proteins • Nucleosome assembly • Storage proteins
*Coffea arabica*	Leaf sections	2DE, MALDI-TOF, MASCOT	Tonietto et al., 2012	
*Gossypium hirsutum*	Hypocotyl sections	iTRAQ, LC-MS/MS, MASCOT	Ge et al., 2014	
		2DE, MALDI-TOF, MASCOT	Zhou et al., 2016	• ROS scavengers • Flavonoids biosynthesis
**NEC or EC vs. SE stages**				
*Gossypium hirsutum*	Hypocotyl sections	iTRAQ, SCX chromatography, LC-MS/MS, Proteome Discoverer-MASCOT	Zhu et al., 2018	• ROS scavengers • Signal transduction molecules • Transcriptional regulation • PGR metabolism/signaling pathways • Polyamine metabolic pathway • Fatty acid biosynthesis/metabolism enzymes
*Medicago truncatula*	Leaf sections	2DE, MALDI-TOF	Imin et al., 2005	Genotypes with contrasting embryogenic potential • Primary metabolism associated enzymes • ROS Scavengers
	Wounded leaflets	2DE, MALDI-TOF, MASCOT	Almeida et al., 2012	
**NEC or SE vs. ZE stages**				
*Cyclamen persicum*	ZE and Embryogenic suspension culture	2DE-DIGE, MALDI-TOF, MASCOT	Rode et al., 2012	• ROS scavengers • Molecular chaperones • Glycolysis • Proteasomal components/factors • PGR metabolism/signaling pathways • Cell wall growth and development • Storage proteins
	ZE: Immature zygote embryo SE: Tuber sections	2DE, MALDI-TOF, MASCOT	Bian et al., 2010	
	Dissected ZE Embryogenic suspension culture	2DE, LC-MS/MS, MASCOT	Winkelmann et al., 2006	
*Theobroma cacao*	Dissected ZE Embryogenic suspension culture	2DE-DIGE, LC-MS/MS, MASCOT	Rode et al., 2011	• ROS scavengers • Molecular chaperones • Glycolysis • TCA • Proteases • Carbohydrate metabolism • Amino acid metabolism
	Immature zygote embryo	2DE, MALDI-TOF, MASCOT	Noah et al., 2013	
*Pinus pinaster*	Immature zygote embryos	1D cut pieces, 2DE, LC-MS/MS, Proteome Discoverer-MASCOT	Morel et al., 2014b	• Molecular chaperones • Proteasome
**Maturation**				
**PGR**				
*Araucaria angustifolia*	Immature female cones	Label-Free, LC-MS/MS, Progenesis QI	Fraga et al., 2016	PGR-supplemented • PIN-like protein • ATP synthase subunit alpha • Terpenoid biosynthesis • Molecular chaperones
*Picea balfouriana*	Immature zygote embryos	iTRAQ, LC-MS/MS, Proteome Discoverer-MASCOT	Li et al., 2015	6-BAP-dependent proliferation • Fatty acid metabolism • Glycolysis • Tyrosine metabolism • Ribosomal proteins • Disease/defense-related proteins • Calcium-dependent protein kinase 3,6
*Phoenix dactylifera*	Dissected ZE Leaf sections	2DE, MALDI-TOF, MASCOT	Sghaier-Hammami et al., 2010	ABA-dependent vigor • Storage proteins • Glycolysis
**Stress-dependent**				
*Carica papaya*	Immature zygote embryos	2DE, MALDI-TOF, MASCOT	Vale et al., 2014	PEG-dependent maturation • Glycolysis • Molecular chaperones • Fatty acid metabolism
*Pinus pinaster*	Immature zygote embryos	2DE, LC-MS/MS	Morel et al., 2014a	Gellan gum-maturation SE • Proteasome • Molecular chaperones • Glycolysis
*Citrus sinensis*	Immature ovules	2DE, MALDI-TOF, MASCOT	Pan et al., 2010	2,4-D inhibited SE • Osmotic stress associated proteins (Calmodulin-binding protein, osmotin-like protein, thaumatin-like protein)
*Sugarcane*	Axillary leafs sections	Label-free, LC-MS/MS, Progenesis QI and SECEST protein databank	Reis et al., 2016	Polyamine-dependent maturation • ROS Scavengers • Storage proteins
	Shoot apical meristem sections	Label-free, LC-MS/MS, Progenesis QI and SECEST protein databank	Heringer et al., 2017	• Methyltransferases • Vesicular trafficking (clathrin heavy chain 1)
	Shoot apical meristem sections	Label-Free, LC-MS/MS, ProteinLynxGlobal Server and SECEST protein databank	Heringer et al., 2015	• PGR •Metabolism/signaling pathways • Transcriptional regulation • Vesicular trafficking • Primary metabolism associated enzymes • Secreted proteins • ROS Scavengers
*Theobroma cacao*	Somatic embryo-derived cotyledons	Label-free, LC-MS/MS, Progenesis QI and SECEST protein databank	Pila Quinga et al., 2018	• Glycolysis • ROS • Proteolytic enzymes
**SE vs. ZE**				
*Phoenix dactylifera*	Dissected ZE Leaf sections	2DE, MALDI-TOF, MASCOT	Sghaier-Hammami et al., 2009	• ATP synthesis • Glycolysis • TCA cycle • Primary metabolism associated enzymes • Storage proteins • Molecular chaperones
*Larix × eurolepis*	Immature zygote embryos	1D or 2DE, LC-MS/MS, PEAKS Studio	Teyssier et al., 2014	• Storage proteins • Molecular chaperones • Proteasome • Glycolysis
*Theobroma cacao*	Flower sections	2DE, LC-MS/MS, ProteinLynxGlobal Server	Niemenak et al., 2015	• Storage protein • Disease/defense-related proteins
*SE*				
*Fraxinus mandshurica*	Immature zygote embryos	2DE, LC-MS/MS, MASCOT	Liu et al., 2015	• Storage protein • Nucleic acid metabolism (helicase)
*Cyathea delgadii*	Sporophyte culture	2DE, LC-MS/MS, MASCOT	Domzalska et al., 2017	• Glycolysis • Molecular chaperones • Fatty acid metabolism • Proteasome
*Oryza sativa*	Dehusked seed	2DE, MALDI-TOF, MASCOT	Yin et al., 2007	• Glycolysis (starch degradation) • ROS scavengers • Molecular chaperones • Storage proteins
Germination				
*Picea asperata*	Immature zygote embryos	iTRAQ, LC-MS/MS, Proteome Discoverer-MASCOT	Jing et al., 2017	Partial desiccation-treated SE (Cotyledonar) • ROS Scavengers •Glycolysis Fatty acid biosynthesis/metabolism enzymes •Photosynthesis-related proteins •Molecular chaperones •Osmotic-stress related proteins •Ribosome Proteasome
		Lysine-acetylated peptides, LC-MS/MS, MaxQuant	Xia et al., 2016	

*^∗^ Protein classes within the groups sorted by frequency.*

**Table 2:** Promising proteomic technologies for SE.

Proteomic technology	Somatic embryogenesis
Histone PTM modifications	Epigenetics
PTM modifications: phosphoproteomics, ubiquitin proteomics, etc.	Signal transduction
Protein-DNA and RNA-protein interactions	Transcriptional regulation
Protein-protein interactome	Signal transduction
Protein-ligand interactome	Signal transduction
Protein complex-RNA	Posttransductional regulation
Organelle proteomics	Metabolism
INTACT cell type-specific	Cell type-specific

The comparative proteomes from contrasting phenotypes presented during SE have assumed that the later stages of SE resemble that of ZE. Leaf explant contains different types of cells that are differentiated upon culture *in vitro*. All plant cells can generate calluses that are undifferentiated cell mass, and all plant cells called embryogenic cells can be dedifferentiated on the path to developing into somatic embryo. However, histologic studies of somatic embryos under development have suggested multiple origins for somatic embryos, such as proper *in vitro* culture, embryogenic cells derived from somatic cells by dedifferentiation or preexisting embryogenic cells, or both (Quiroz-Figueroa et al., 2002; Yang and Zhang, 2010; Campos et al., 2017). The multiple origins of somatic embryos provide a challenge not only for the over-mentioned deciphering somatic cell conversion to embryogenic cell but also for the detection of low abundance proteins in the complex sample from *in vitro* culture, since most, if not all, if not all, proteomic samples contain both types of plant cells; those cells are either undergoing the embryo formation or never give rise to an embryo.

A variety of explants have been used to study the proteome during the induction of SE, including hypocotyl and leaf sections, immature zygote embryos, unpollinated ovules, shoot meristems, ZE sections, and anther tissue. 2DE is the most exploited proteomic approach followed by 2DE-DIGE, and more recently by techniques such as label-free proteomics, iTRAQ, or antibody-based proteomics approaches (Xia et al., 2016). PGR metabolism and signaling, the ROS scavengers, primary and secondary metabolism associated enzymes, transcription regulation, signal transduction, disease/defense-related proteins, molecular chaperones, proteolysis, and proteasomal component/factors have been identified in SE proteomes. Remarkable ubiquitin-like and ubiquitin protein modification cascade enzymes have become of substantial interest as they regulate PGR signaling in plants (Kelley, 2018).

## Metabolic Pgr-Precursors

Some of the proteins identified in the previous section participate in the metabolism of most, if not all, PGRs or in particular metabolic PGR-precursor synthesis pathways. For instance, cytochrome P450 proteins participate in the metabolism of most PGRs (Werck-Reichhart et al., 2002); phospho-2-dehydro-3-deoxyheptonate aldolase 1 and 3-dehydroquinate synthase (Domzalska et al., 2017) are proteins that link the glycolysis and the pentose phosphate pathways and lead to the synthesis of phenylalanine and tryptophan as well as secondary metabolites. Indole-3-acetic acid (IAA), the main PGR with a role in the growth and development process, is synthesized mainly from tryptophan as a metabolic precursor (Zhao, 2012). Methionine synthase and *S*-adenosylmethionine synthetase are key enzymes in the synthesis of methionine and the metabolic precursor *S*-adenosylmethionine (*S*-AdoMet) from aspartate, and act by feeding ethylene and polyamine production within the cell (Ravanel et al., 1998); more recently, they have been associated with the DNA methylation system (Morel et al., 2014b; De-la-Peña et al., 2015). These are detected as more abundant at early SE in the fern *Cyathea delgadii* (Domzalska et al., 2017), *Zea mays* (Sun et al., 2013), *P. glauca* (Lippert et al., 2005), *Citrus sinensis* (Pan et al., 2009), *Pinus nigra* (Klubicová et al., 2017), and *Persea americana* (Guzmán-García et al., 2013), and more abundant in somatic embryo maturation stages in *Quercus suber* (Gomez-Garay et al., 2013), *Pinus pinaster* (Morel et al., 2014b), *Araucaria angustifolia* (Jo et al., 2014), and *Larix × eurolepis* (Teyssier et al., 2011, 2014). Aspartate aminotransferase, an aminotransferase enzyme that catalyzes the interconversion of aspartate and α-ketoglutarate by transferring the amino group and yields oxaloacetate and glutamate, was detected as more abundant during the late stages of somatic embryo development in *Medicago truncatula* and in *C. delgadii* (Almeida et al., 2012; Domzalska et al., 2017). The 2,3-bisphosphoglycerate-independent phosphoglycerate is an enzyme that participates in the glycolysis pathway and was found to be more abundant in EC.

## Proteins Related to Synthesis and Metabolism of PgrS

Because many proteins that participate in the synthesis, degradation, transport, perception, and signaling of PGRs have been identified in many plant species, an array of biosynthesis pathways have been proposed, from inter and intracellular transport to many degradative pathways, receptors, and signaling-related proteins for PGR molecules. However, how the number of proteins in those pathways is differentially expressed has not been reported, maybe as a result of the action of either transcriptional, posttranscriptional, or posttransductional regulatory mechanisms, the natural low abundance of certain proteins (e.g., transcription factors), or organelle-dependent enzyme localization (e.g., chloroplast). One notable exception are the proteins seen in proteomic studies using iTRAQ in *Gossypium hirsutum*, *Musa* spp. AAA cv. Grand Naine, and *Z. mays* (Ge et al., 2017; Kumaravel et al., 2017; Zhu et al., 2018). Those proteins include indole-3-pyruvate monooxygenase protein YUCCA3, an enzyme of the auxin biosynthesis, in *Musa* spp. AAA cv. Grand Naine that is more abundant in EC (Kumaravel et al., 2017), and an IAA-conjugate hydrolase GH3 that is more abundant in both EC and late SE stages in *Z. mays* and *G. hirsutum* (Ge et al., 2017; Zhu et al., 2018). GH3 collectively may result in an increase in IAA (Zhao et al., 2001). Adenylate isopentenyltransferase in *Musa* spp. AAA cv. Grand Naine and cytokinin trans-hydroxylase are upregulated in the EC of *Z. mays* (Ge et al., 2017; Kumaravel et al., 2017), cytokinin receptors CRE1 and B-ARR are more abundant in EC, and histidine phosphotransfer protein is upregulated in SE in *G. hirsutum* (Zhu et al., 2018), factors that are required for the cytokinin response (Mason et al., 2005). Phospholipase A1, 12-oxophytodienoic acid reductase, acyl-CoA oxidase, and enoyl-CoA hydratase/3-hydroxyacyl-CoA dehydrogenase are more abundant in EC at the II stage (Ge et al., 2017). These are collectively required for the jasmonic acid biosynthesis-wound-dependent pathway (Turner et al., 2002). Arogenate dehydrogenase, involved in the salicylic acid synthesis, is upregulated in the maturation stages of the EC stage in *Z. mays* (Ge et al., 2017).

Abscisic acid plays an essential role in the accumulation of nutritive products during the development and maturation of somatic embryos (Jin et al., 2014). Pyrabactin resistance/Pyrabactin resistance-like and ABRE-binding factor transcription factor, abscisic acid signal pathway proteins from NEC to somatic embryo stages, were upregulated in EC and then downregulated in somatic embryo stages. The level of Pyrabactin resistance/Pyrabactin resistance-like showed no significant difference between NEC and EC, and was upregulated in somatic embryo stages compared with EC in *G. hirsutum* (Zhu et al., 2018).

The role of ethylene in embryogenic induction is complicated by its inconsistent effects on different plants and culture systems. Constitutive triple response-1 protein, which is involved in the ethylene signal pathway, was slightly downregulated in somatic embryo stages compared to EC, suggesting a possible negative role of Constitutive triple response-1 in *G. hirsutum* somatic embryo maturation (Zhu et al., 2018). Gibberellin-insensitive dwarf-1 protein, a gibberellin receptor, is upregulated from NEC to EC and unchanged in the somatic embryo vs. EC. DELLA is unchanged from NEC to EC and downregulated in the somatic embryo in *G. hirsutum* (Zhu et al., 2018). The cytochrome P450 734A1/PhyB-4 activation-tagged suppressor1 upregulated protein in EC in *Z. mays* (Ge et al., 2017) leads to the accumulation of inactive brassinolide 26-hydroxy-brassinolide (Neff et al., 1999), and is downregulated in brassinolide perception as brassinosteroid-insensitive-1 is downregulated and BRI1-associated receptor kinase1 is upregulated in EC and NEC and the corresponding somatic embryo stages in *Z. mays* and *G. hirsutum* (Ge et al., 2017; Zhu et al., 2018).

## Glycolysis-Supplied Energy Leads Embryo Formation

Glycolysis, a key and ubiquitous metabolic pathway by which the plant cell converts carbohydrates to the energetic coin ATP, is a central pathway to generate energy and metabolic intermediaries that sustain the biosynthesis of intra- and extra-cellular molecules required by the cell. Proteins of the glycolysis pathway such as phosphofructokinase, fructose-1,6-biphosphate aldolase, glyceraldehyde-3-phosphate dehydrogenase, triosephosphate isomerase, phosphoglycerate mutase, 2,3-bisphosphoglycerate-independent phosphoglycerate mutase, phosphoglycerate kinase, enolase, and pyruvate decarboxylase were reported to be more abundant in the EC stage of African oil palm (Silva et al., 2014), *G. hirsutum* (Ge et al., 2017), maize (Sun et al., 2013; Varhaníková et al., 2014; Ge et al., 2017), saffron (Sharifi et al., 2012), *Cyphomandra betacea* (Correia et al., 2012b), Musa (Kumaravel et al., 2017), *Vitis vinifera* (Zhang et al., 2009), *Larix principis-rupprechtii* (Beversdorf, 1987; Zhao et al., 2015a), *A. angustifolia* (dos Santos et al., 2016), *P. nigra* (Klubicová et al., 2017), *Theobroma cacao* (Niemenak et al., 2015), and sugarcane (Heringer et al., 2017).

Proteomic studies employing ZE as a reference also showed that the enzymes of the glycolytic pathway are more abundant in the callus- and explant-derived SE stages from *Q. suber* (Gomez-Garay et al., 2013), *C. delgadii* (Domzalska et al., 2017), *M. truncatula* (Almeida et al., 2012), sweet orange (Pan et al., 2009, 2010), *Coffea arabica* (Tonietto et al., 2012), *Cyclamen persicum* (Winkelmann et al., 2006), *G. hirsutum* (Ge et al., 2014), *Q. suber* (Gomez-Garay et al., 2013), *L. principis-rupprechtii* (Zhao et al., 2015a), papaya (Vale et al., 2014), *Larix × eurolepsis* (Teyssier et al., 2011, 2014), *P. glauca* (Lippert et al., 2005), date palm (Sghaier-Hammami et al., 2010), *C. persicum* Mill (Winkelmann et al., 2006), and *T. cacao* (Noah et al., 2013). These findings indicate that the glycolysis pathway is used both by cells undergoing dedifferentiation and the somatic embryo, driving growth and the development process.

It is unknown how the existing cytosolic and/or chloroplastic glycolysis pathways’ rate flux changes during SE to balance between energy production by the mitochondria and energy use that occurs as metabolic intermediaries are fed to the other biosynthetic pathways, such as fatty acid and secondary metabolites, operating in many cell organelles. Some proteins related to the glycolysis pathway likely regulate glycolysis by enhancing respiration or photosynthesis. Pyruvate dehydrogenase complex, dihydrolipoyl acetyltransferase, and dihydrolipoyl dehydrogenase are accepted as potential control points for metabolism, moderating the balance between catabolism and anabolism. This makes sense, given their connection with other metabolic pathways, and the fact that it is tightly regulated by multiple factors, including light, product inhibition, organelle-specific mechanism, and phosphorylation/dephosphorylation (Luethy et al., 2001; Tovar-Méndez et al., 2003).

Chloroplast and mitochondrial α and β isoforms of pyruvate dehydrogenase E1 from *C. persicum* (Lyngved et al., 2008) and *C. delgadii* (Domzalska et al., 2017) were found to be upregulated in EC as well in somatic embryo maturation. Research indicates the chloroplast dihydrolipoyl dehydrogenase 1 is upregulated from *C. delgadii* (Domzalska et al., 2017), *Larix × eurolepis* (Teyssier et al., 2014), and *P. nigra* (Klubicová et al., 2017). Furthermore, pyruvate dehydrogenase was downregulated in ZE from *C. persicum* (Mwangi et al., 2013). Whether the regulation of pyruvate dehydrogenase protein by phosphorylation is occurring during the SE process or if this protein is regulated by unknown mechanisms is still not known.

## Fatty Acids

Acetyl-CoA derived from glycolysis by the action of the pyruvate dehydrogenase complex functions as a link between carbon metabolism and fatty acid biosynthesis. As a substrate of the acetyl-CoA carboxylase (ACC), acetyl-CoA is used to produce malonyl-CoA, then utilized as a malonyl group donor, which is transferred to the acyl-carrier protein (ACP) by a malonyl-CoA:acyl carrier protein, malonyltransferase. The fatty acid biosynthesis pathway involves a multienzymatic cascade by the action of 3-ketoacyl-ACP synthase (KAS) isoforms I, III, and II, 3-ketoacyl-ACP reductase, hydroxyacyl-ACP dehydratase (HAD), and enoyl-ACP reductase (ENR) that utilizes acetyl-CoA as the starting unit and malonyl-CoA as a two-carbon unit donor to yield an 18-carbon fatty acid attached to ACP. Then, the saturated or desaturated fatty acids released from the fatty acid machinery by an ACP desaturase (SAD) and/or acyl-ACP thioesterase can either enter the galactolipid, sulfolipid, and/or phospholipid synthesis pathways or be exported from the plastid to the ER, where they ultimately coalesce in oil droplets of fat to wax and/or cutin synthesis (Mou et al., 2000; White et al., 2005; Pidkowich et al., 2007; Li-Beisson et al., 2013).

The proteome from early SE of *C. delgadii* (Domzalska et al., 2017) and maturation stages of the somatic embryo from *P. pinaster* (Morel et al., 2014a) exhibited ACC as an upregulated protein; an accumulation of this protein has also been seen in biochemical assays as an increase in the activity of the ACC enzyme in EC and somatic embryos from carrot (Wurtele and Nikolau, 1992). Malonyltransferase, as well as KAS I, II, and II, was upregulated in both EC and somatic embryos from *G. hirsutum* (Zhu et al., 2018). Enoyl-ACP reductase was upregulated in the maturation of polyethylene glycol (PEG)-dependent somatic embryo from *C. papaya* (Vale et al., 2014) and even in the ZE from *C. persicum* (Mwangi et al., 2013). Additionally, SAD was detected in EC from *C. persicum* (Lyngved et al., 2008).

The upregulation of proteins of the core fatty acid biosynthesis pathway in early somatic embryo formation and somatic embryo maturation suggests that this central pathway is active and might support downstream metabolic pathways required during SE. Lipid transfer proteins, a collection of extracellular proteins with a secretory peptide, are thought to participate in the movement of surface lipids required for the formation of wax and cutin (Wirtz, 1991; Li-Beisson et al., 2013). A lipid transfer protein was upregulated in CSE from *G. hirsutum* (Ge et al., 2014) and phospholipid transfer protein 1 was upregulated during somatic embryo maturation of sugarcane embryos in a light quality-dependent fashion (meaning embryo maturation under white light plus medium blue, red, and far-red) (Heringer et al., 2017), suggesting that surface lipids are an essential factor in embryo maturation.

To utilize fatty acid as a source of energy or to generate metabolic precursors, plants have exploited an array of lipases and the β-oxidation pathway. Remarkably, SE process proteomic studies so far have found Gdsl esterases/lipases to be upregulated in EC from *A. angustifolia* (dos Santos et al., 2016), in proembryonic masses from in *P. americana* (Guzmán-García et al., 2013), in the SE cotyledonar stage of *G. hirsutum* (Ge et al., 2014), and in PEG-dependent SE maturation in *C. papaya* (Vale et al., 2014). Nevertheless, the physiological role of Gdsl esterases/lipases in SE is still mostly undetermined. Numerous members of the Gdsl esterases/lipases have been well documented in plants, and their participation in signal transduction during plant development, the synthesis of secondary metabolites, and plant defense response have been well studied, including Arabidopsis Gdsl lipase 2 (Lee et al., 2009), which is very attractive for studying them during the induction of SE. Core proteins of β-oxidation process such as CoA oxidase (ACX) in EC from *Z. maize* (Ge et al., 2017), and the multifunctional protein in both EC from *Z. maize* and SE maturation of *Larix × eurolepsis* were found to be upregulated (Teyssier et al., 2014; Ge et al., 2017). The upregulation of core proteins in a wide range of conserved metabolic pathways such as fatty acid biosynthesis and β-oxidation process suggests a tight regulation between those processes, particularly in the EC, but also at early stages of somatic embryo development, as well as in somatic embryo maturation.

## Ros and Counterpart Ros Scavengers During Somatic Embryogenesis

Reactive oxygen species [ROS, reviewed in Das and Roychoudhury (2014)] are side products of the aerobic metabolism in an oxygen-evolving photosynthetic organism (Halliwell, 2006). ROS can be free radicals such as O2^−^ and OH, as well as non-radicals such as H_2_O_2_ and ^1^O_2_. As counteractive mechanisms, plants have adopted a sophisticated battery of ROS scavengers to reduce the harmful effect of ROS in the cell. It is accepted that ROS production by a stress stimuli might be required to induce cell dedifferentiation, triggered by the mechanical damage of an explant and/or stimulated by a variety of molecules such as the auxin analog 2,4-dichlorophenoxyacetic acid (2,4-D) and PEG (Fehér, 2015). Proteins related to detoxification include superoxide dismutase in *P. Americana* (Guzmán-García et al., 2013), *Q. suber* (Gómez et al., 2009), *V. vinifera* (Zhang et al., 2009), and *L. principis-rupprechtii* (Zhao et al., 2015a); ascorbate peroxidase in *Z. mays* (Sun et al., 2013); glutathione-*S*-transferase in *C. sativus* (Sharifi et al., 2012), *V. vinifera* (Marsoni et al., 2008), and *M. truncatula* (Imin et al., 2005); catalase (CAT) in *C. persicum* (Rode et al., 2012) and *L. principis-rupprechtii* (Zhao et al., 2015b); and monodehydroascorbate reductase and dehydroascorbate reductase in *A. angustifolia* (dos Santos et al., 2016). All of these enzymes are upregulated in EC. Therefore, an active counteraction to ROS by ROS scavengers is an important factor in the fate of EC.

In contrast to EC, early somatic embryo initiation displayed downregulation of some proteins related to detoxification, such as Mn superoxide dismutase in *G. hirsutum* (Ge et al., 2014); CAT in *C. delgadii* (Domzalska et al., 2017), and *Q. suber* (Gomez-Garay et al., 2013); ascorbate peroxidase in *Q. suber* (Gomez-Garay et al., 2013) and *G. hirsutum* (Ge et al., 2014); and guaiacol peroxidase (GPX) in *G. hirsutum* (Ge et al., 2014), although this enzyme is upregulated across PGR- or stress-dependent somatic embryo maturation (Teyssier et al., 2011; Morel et al., 2014a; Vale et al., 2014; Li et al., 2015). Notably, at the cotyledonar somatic embryo stage, GPX is even more abundant at the somatic embryo stage (Rode et al., 2012) than its ZE counterpart (Gomez-Garay et al., 2013; Mwangi et al., 2013; Morel et al., 2014b; Silva et al., 2014; Niemenak et al., 2015).

Moreover, the upregulation of CAT has been detected in the partial desiccation treatment of germinated somatic embryo of *P. asperata* (Jing et al., 2017). Accurate quantification by iTRAQ has allowed the unraveling of two CAT3 and one CAT1 upregulated proteins in GSE and CSE compared with their proembryonic masses, and one CAT1 is upregulated in GSE followed by downregulation in the CSE in *L. principis-rupprechtii* (Zhao et al., 2015a). The presence of multiple protein isoforms might be an indication that posttranscriptional regulation mechanisms are essential during the induction of SE.

## Molecular Chaperones

The myriad of newly synthesized proteins, as well as folded proteins that can either develop into the unfolded form or coalesce into aggregates upon stress exposure, that require a folding process to develop into biologically active proteins is significant (Boston et al., 1996). Therefore, the active folding process that is mainly determined in the primary sequence and enhanced by the action of foldases or molecular chaperones is crucial for the proper function of the cell. The foldase enzymes, such as protein disulfide isomerase that catalyzes the arrangement of disulfide bonds and peptidyl prolyl isomerase (PPI) that switches the conformation of the peptide bond prior to a proline residue, participate in refolding processes. In addition to their refolding role in the cell, chaperones such as heat shock proteins (HSP) also have a role in preserving proteins in an unfolded form that is suitable for translocation of proteins across membranes, evading the formation of protein aggregates, enabling disassembly of aggregated proteins (Ellis and Van der Vies, 1991), and, as recent research shows, participate in degradation of protein complexes and even cell organelles under stress conditions (Marshall and Vierstra, 2018).

Peptidyl prolyl isomerase was found to be upregulated in EC (Zhao et al., 2015b), downregulated during proembryogenic masses’ GSE, then upregulated in CSE in *L. principis-rupprechtii* (Zhao et al., 2015a). During the maturation of the somatic embryo, PPI was detected as unique for a somatic embryo derived from EC and upregulated in a mutual white/blue/red/far-red-dependent maturation of sugarcane (Heringer et al., 2015, 2017), upregulated in GSE of *Larix × eurolepsis* (Teyssier et al., 2014), the CSE stage of the somatic embryo in *C. persicum* Mill (Rode et al., 2012), and shown to be more abundant in somatic embryo maturation than ZE maturation of *T. cacao* (Niemenak et al., 2015).

In addition, FKBP-type PPI was found to be upregulated in the CSE of *G. hirsutum* (Ge et al., 2014) and in somatic embryos undergoing maturation in *M. truncatula* (Almeida et al., 2012). A cyclophilin-type PPI was detected to be upregulated in GSE and CSE of *C. arabica* (Tonietto et al., 2012). As cyclophilins are encoded by multiple genes (Romano et al., 2004), and might be a part of a conserved evolutionary mechanism that assists in the expression of the auxin-regulated genes (Lavy and Estelle, 2016), further experimentation regarding cyclophilin’s role during SE is needed. Both of the ATP-dependent chaperones HSP70 and HSP100 were detected in somatic embryos, in contrast to the CplA/HSP100 that was upregulated in EC of *Z. mays* (Varhaníková et al., 2014).

Variants of the HSP70 were detected extensively among the SE plant models. HSP70 was found to be uniquely expressed in EC from *Musa* spp. AAA cv. Grand Naine (Kumaravel et al., 2017) and rice (Yin et al., 2007). Moreover, HSP70 is upregulated in the callus of *C. persicum* (Rode et al., 2012), and in the EC of *C. betacea* (Correia et al., 2012a), *V. vinifera* (Marsoni et al., 2008), and *Crocus sativus* (Sharifi et al., 2012). Variants of HSP are upregulated in callus- and explant-derived somatic embryo stages that include GSE in *P. glauca* (Lippert et al., 2005) and *C. delgadii* (Domzalska et al., 2017), and in polyamine-dependent maturation of somatic embryo from sugarcane (Reis et al., 2016) and in PEG-dependent somatic embryo maturation in *C. papaya* (Vale et al., 2014). Similar expression patterns, upregulated between proembryogenic masses and GSE stages and followed by downregulation during CSE in *L. principis-rupprechtii*, have been reported (Zhao et al., 2015a,b).

HSP70 was also detected in ZE of *T. cacao* (Niemenak et al., 2015), and was more abundant in somatic embryos than in the ZE counterpart in *C. persicum* (Winkelmann et al., 2006). Findings in *Phoenix dactylifera* that displayed HSP70 as more abundant in ZE than in SE (Sghaier-Hammami et al., 2009) suggest that both up and downregulation of HSP70s are occurring during SE. Indeed, HSP70 belongs to multiple gene families. HSP90 was upregulated during the induction of SE in *Elaeis guineensis* (Silva et al., 2014) and in *C. delgadii* (Domzalska et al., 2017), downregulated during SE in samples containing somatic embryo stages from globular to cotyledonar in *G. hirsutum* (Zhu et al., 2018), and upregulated in *Larix × eurolepsis* under gellan gum-dependent maturation at the SEG (Teyssier et al., 2011) and cotyledonar stages (Teyssier et al., 2014). Bip/GRP78 were found to be upregulated between proembryogenic masses and GSE stages followed by downregulation during CSE in *L. principis-rupprechtii* (Zhao et al., 2015a), and in PEG-dependent maturation in *C. papaya* (Vale et al., 2014).

The chaperonin HSP60 was detected as downregulated in the EC of *C. persicum* (Lyngved et al., 2008), upregulated in the cotyledonar stage of both somatic embryo and ZE-derived embryos from *G. hirsutum* (Ge et al., 2014) and the cotyledonar stage of *C. persicum* (Mwangi et al., 2013). The small HSP class I chloroplast HSP 25.3 was found to be uniquely expressed in EC from *Musa* spp. AAA cv. Grand Naine (Kumaravel et al., 2017), and the cytosolic HSP type 2 was upregulated at the induction of SE in *C. delgadii* (Domzalska et al., 2017). During the induction of SE in *P. pinaster*, the cytosolic HSP18.2 was upregulated in CSE and the counterpart in the ZE-derived embryo, and the class II HSP17.6 was upregulated during somatic embryo maturation (Morel et al., 2014b).

## Proteolytic Enzymes and the Ubiquitin Proteasome System

Biochemical analyses of the proteolytic activity reveal that serine proteases, aspartic proteases, and metalloproteases are dominant in EC, whereas serine proteases dominate NEC in *Solanum betaceum* (Alves et al., 2017). Leucine aminopeptidase has been found to be unique to PEG-dependent somatic embryo maturation of *C. papaya* (Vale et al., 2014). Metalloprotease m41 and aspartyl protease have been detected as upregulated in EC of *A. angustifolia* and in the blue/red light-dependent somatic embryo maturation in sugarcane (dos Santos et al., 2016; Heringer et al., 2017), downregulated at somatic embryo maturation in cacao (Pila Quinga et al., 2018) and at the torpedo stage of SE compared to the torpedo stage of ZE (Noah et al., 2013; Niemenak et al., 2015). The cysteine protease displays a similar pattern: this enzyme is upregulated at the early stages of SE in *P. glauca* (Lippert et al., 2005) and downregulated during the somatic embryo maturation in cacao (Pila Quinga et al., 2018).

Intriguingly, evidence of accumulation of cystatin, a cysteine protease inhibitor, in the EC of saffron (Sharifi et al., 2012) and *Vigna unguiculata* (Nogueira et al., 2007) has been reported. Therefore, protease action is very dynamic and linked to the counteractive action of proteolytic inhibitors during the SE process.

Ubiquitin (Ub) and proteins related to Ub, also called ubiquitin-like proteins, are attached to substrates via a cascade of related enzymes E1→E2→E3; then these modified proteins face a selective and non-selective degradation into the 26S proteasome. Also, ubiquitination directs protein trafficking or modified protein properties as well as location in the cell (Vierstra, 2009; Marshall and Vierstra, 2018).

Ub and poly-Ub 10 have been detected in embryogenic cell suspension of cowpea (Nogueira et al., 2007) and in explants undergoing SE in cassava (Baba et al., 2008); both are upregulated in GSE and CSE in *L. principis-rupprechtii* (Zhao et al., 2015b). Poly-Ub 11 was upregulated 61-fold in somatic embryo polyamine-dependent maturation (Reis et al., 2016). Ub fused to the ribosomal protein S27a in embryogenic suspension culture of *V. unguiculata* (Nogueira et al., 2007), in the EC and NEC stages of *Z. mays* (Ge et al., 2017), and during the induction of SE in cassava (Almeida et al., 2012). Poly-Ub 11 is upregulated in the embryogenic callus of *C. persicum* (Rode et al., 2012). Interestingly, the constitutive overexpression of S27a has shown a correlation with the increase of proliferation of undifferentiated cells and arrest of the shoot and leaf development (Hanania et al., 2009).

Small ubiquitin-related modifier (SUMO), an ubiquitin-like protein, has been found to be upregulated in GSE and CSE in *L. principis-rupprechtii* (Zhao et al., 2015a), and during PGR- or PEG-dependent somatic embryo maturation (Vale et al., 2014; Fraga et al., 2016). Interestingly, the disruption of the two encoded *SUMO* genes in *A. thaliana* results in an early embryo lethal phenotype (Saracco et al., 2007). Ubiquitin-conjugated enzymes (called E2/UBC) have been identified; UBC5 in leaf-derived somatic embryos of *C. delgadii* (Domzalska et al., 2017), Ub carrier protein E235 in somatic embryos of cacao (Niemenak et al., 2015), the E2 variant 1D/MMS2 during somatic embryo maturation, UBC32 during polyamine-dependent somatic embryo maturation, and UBC9, which is downregulated in blue/red light-dependent somatic embryo maturation of sugarcane (Reis et al., 2016; Heringer et al., 2017). SUMO-activating enzyme subunit 2-like/SAE2 is upregulated during the maturation of somatic embryo of *P. pinaster* (Morel et al., 2014a) and in the blue/red light-dependent maturation of somatic embryo of sugarcane (Heringer et al., 2017).

E3 ubiquitin ligase is responsible for recognizing ubiquitination targets across the cell. In the EC of sugarcane and *A. angustifolia*, the RING-type E3 ubiquitin ligases BRCA1 and ORTH2 were identified, respectively (Heringer et al., 2015; dos Santos et al., 2016), while during somatic embryo maturation the RING-type ubiquitin ligase ARI2 is upregulated in *A. angustifolia* (Fraga et al., 2016).

The components of the SCF-(Skp1-CULLIN1-F-box)-type Ub ligase such as an F-box protein and a SKP1 protein were found to be upregulated during maturation in either *C. sinensis* or *A. angustifolia* (Pan et al., 2010; Fraga et al., 2016).

The proteasome that executes the degradation of ubiquitinated proteins comprises the catalytic core particle (CP), where proteolysis occurs, and two terminal regulatory particles (RP) that serve as the switch for the activation of the proteasome. CP proteins such as PAA1 were detected in an embryogenic cell suspension of *V. unguiculata* and found to be upregulated in the EC of *V. vinifera* and from CSE to the mature somatic embryo of *Q. suber* (Nogueira et al., 2007; Zhang et al., 2009; Gomez-Garay et al., 2013). PBA1 was detected in embryogenic suspension cultures of *V. unguiculata* and found to be upregulated in the somatic embryo of *P. pinaster* and in *Q. suber* from CSE to the mature somatic embryo (Gomez-Garay et al., 2013; Morel et al., 2014b). PBF1 is upregulated in somatic embryo gellan gum-dependent maturation of *P. pinaster* (Morel et al., 2014a,b).

Regulatory particles proteins, such as RPT5, are upregulated in the EC of *C. persicum*, *Musa* spp. AAA cv. Grand Naine, saffron and H99 inbred maize (Lyngved et al., 2008; Sharifi et al., 2012; Sun et al., 2013; Kumaravel et al., 2017), and have been identified in the somatic embryo and ZE of *C. persicum* (Bian et al., 2010). Additionally, RPT1 is upregulated during the SE of *C. delgadii* (Domzalska et al., 2017). RPT3 is found in the early development stages of SE in *C. persicum* (Rode et al., 2012), and RPT2 is found in the somatic embryo gellan gum-dependent maturation of *P. pinaster* (Morel et al., 2014a). RPN12 is upregulated during somatic embryo maturation in *C. delgadii* and NEC of *Musa* spp. AAA cv. Grand Naine (Domzalska et al., 2017; Kumaravel et al., 2017). Interestingly, the disruption of *RPN12A* in *A. thaliana* results in a decrease in its sensitivity to cytokinins and 2,4-D; given the upregulation of cytokinin-inducible genes such as CYCD3 and NAI1 in *rpt12a* seeds, the authors suggest that a feedback-inhibitory mechanism is present (Smalle et al., 2002). Recently, it was shown that this inhibition requires the response regulator B-type ARR5 that is accumulated in the *rpn12a* seeds (Jasmina et al., 2013). RPN9 is upregulated in blue/red light-dependent maturation of the somatic embryo of sugarcane (Heringer et al., 2017). Collectively, the differential expression of Ub, Ub-like modifiers, components of the Ub cascade, and 26S proteasome suggests the participation of Ub-dependent protein degradation during the SE process.

## Conclusion and Perspectives

**FIGURE 4:**
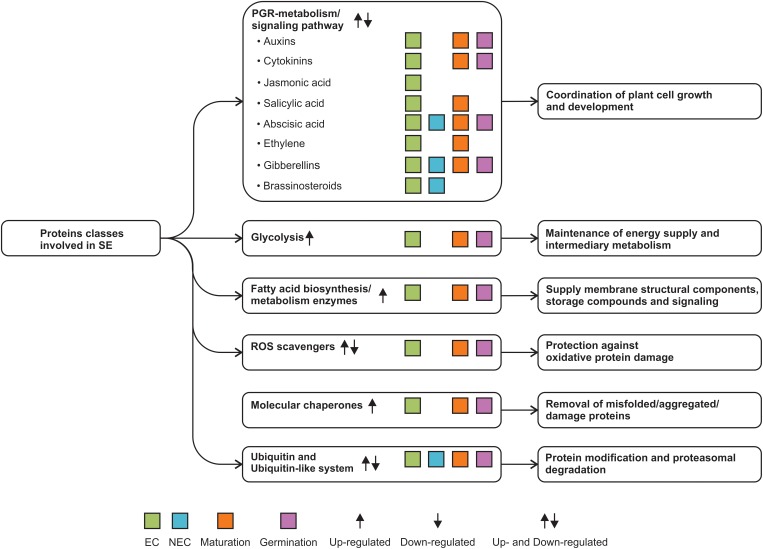
SE proteome data reveal new protein classes involved in the SE process.

Proteomic approaches are emerging as a powerful tool to define the somatic embryo’s changes through the early stages of its development and during the maturation and germination of somatic embryos. As proteomic studies in other systems, SE proteomics studies need to cope with factors such as protein abundance and sample complexity that limit to performer dipper proteome analysis. Protein concentration into the cell is a key factor related to protein abundance and sample complexity, which also limits a dipper proteome analysis of the SE process since somatic embryo can come from multiple origins and the abundance of proteins with a role in somatic embryo formation could be spatiotemporal-dependent. Most current studies have examined the early stages of somatic embryo development using EC and NEC as the model, along with PGR-, PEG-, and blue/red light-dependent somatic embryo maturation. Proteomics is helping to decipher the signal that switches the genetic program from a somatic cell to an embryogenic cell and its later conversion to a mature embryo. Glycolysis, fatty acid biosynthesis, ROS scavengers, and molecular chaperones are the most reiterative protein classes identified by proteomes of SE. However, there are still a large number of important metabolic pathways to study, such as ATP metabolism and the mechanism of signal transduction for auxins and cytokinins. No less critical could be to explore protein modifications via ubiquitin and ubiquitin-like and their connection with molecular chaperones protein class to control the abundance of key proteins for SE, mostly unknown so far (Figure 4).

Finally, it is essential to turn to the study of the organelle proteome during SE. The isolation of the nucleus, mitochondria, vacuoles, and other organelles can lead to the discovery of key proteins involved in the process of induction of SE. It is of particular interest to use the new quantitative proteomic techniques, since in many cases it is not the absence/presence of a protein that determines the physiological effect, but its amount. It is also essential to determine the patterns of modification in the proteins, i.e., acetylation, phosphorylation, ubiquitination, and many other protein modifications. All of these techniques (Table 2), together with the transcriptomics studies, will achieve new insights into the understanding of the dynamic and complex interconnection of events that take place during the induction of somatic embryogenesis.

## Author Contributions

All authors developed the idea and drafted the manuscript.

## Conflict of Interest Statement

The authors declare that the research was conducted in the absence of any commercial or financial relationships that could be construed as a potential conflict of interest.
